# Protective effects of flavonoids against silver nanoparticles-induced toxicity

**DOI:** 10.1007/s00204-025-04068-2

**Published:** 2025-06-10

**Authors:** Inês Santos, Adelaide Sousa, Abel Vale, Félix Carvalho, Eduarda Fernandes, Marisa Freitas

**Affiliations:** 1 LAQV, REQUIMTE, Laboratory of Applied Chemistry, Department of Chemical Sciences, Faculty of Pharmacy, University of Porto, Rua de Jorge Viterbo Ferreira n.º 228, 4050-313 Porto, Portugal; 2 UCIBIO‑Applied Molecular Biosciences Unit, Laboratory of Toxicology, Department of Biological Sciences, Faculty of Pharmacy, University of Porto, 4050‐313 Porto, Portugal; 3 Associate Laboratory i4HB‑Institute for Health and Bioeconomy, Faculty of Pharmacy, University of Porto, 4050‑313 Porto, Portugal

**Keywords:** Silver nanoparticles, Toxicity, Flavonoids, Antioxidant, Anti-inflammatory

## Abstract

Silver nanoparticles (AgNP) are becoming increasingly prevalent in daily life due to their unique properties, which have expanded their application across multiple sectors. This widespread use has led to a marked rise in human exposure to AgNP, raising concerns about their safety and potential health impacts. Studies have demonstrated that AgNP can induce harmful effects, including oxidative stress and pro-inflammatory responses, underscoring the need to identify protective agents to mitigate health risks. Flavonoids, known for their anti-inflammatory and antioxidant properties, hold significant  promise as effective agents in mitigating the toxic effects of AgNP. This review examines the current literature on the protective effects of flavonoids against AgNP toxicity. It highlights the underlying mechanisms by which flavonoids exert protective actions, with a focus on relevant pathways and molecular interactions. The results of in vitro and in vivo studies demonstrated that flavonoids exert protective effects against AgNP-induced damages through their antioxidant and anti-inflammatory activity. This analysis underscores the flavonoids potential as a promising strategy to reduce the negative impacts of AgNP, supporting safer and more sustainable applications of nanotechnology across diverse fields.

## Introduction

Silver nanoparticles (AgNP) are inorganic nanoparticles (NP) composed of metallic silver, valued for their unique physicochemical properties that support a wide range of applications in medicine, electronics, and environmental science (Abbasi et al. 2016; Zhang et al. 2016). Their extensive utility stems from their biological activities, including antibacterial, antifungal, antiviral, and anticancer effects, making them suitable for various applications (Sharma 2008; Zhang et al. 2016). AgNP are also used in several pharmaceutical formulations, such as burn ointments, and can be biofunctionalised for targeted drug delivery by incorporating biomolecules to enhance specificity (Austin et al. 2014). The primary driver of their wide usage is their antimicrobial action, which finds applications across multiple fields. In medicine, AgNP are incorporated into wound dressings, antiseptic sprays, and as coatings for catheters (urinary and venous) (Burdu et al. 2018; Nie et al. 2023). In the agricultural sector, AgNP play a role in enhancing productivity, especially through their application as nanopesticides and nanofertilizers (Khan et al. 2023; Mehmood 2018). Furthermore, AgNP also have an important role in food preservation and packaging, thereby extending the shelf life and preventing the deterioration of foodstuffs (Ashfaq et al. 2022; Biswas et al. 2022; Zorraquín-Peña et al. 2020). Furthermore, the textile industry uses AgNP to coat fabrics (e.g., cotton), primarily due to their antimicrobial properties, as there is a growing demand for multifunctional fabrics that promote both health and hygiene (Maghimaa and Alharbi 2020; Ribeiro et al. 2022). Consequently, AgNP are increasingly embedded into an expanding range of everyday products.

As a consequence of the several applications of AgNP, an increased use in our daily lives results in heightened and inadvertent exposure, which has led to heightened concerns regarding their safety and the possible impact on human health and the natural environment (Ferdous and Nemmar 2020). The exposure to this NP can occur through different forms, including inhalation, ingestion, dermal absorption, and injection, where the oral route has become one of the most significant (Vitulo et al. 2022). Once AgNP enter the human body, cellular uptake can occur through endocytosis, diffusion, or paracellular pathways, with endocytosis being the most common (Imai et al. 2017). However, the uptake process is significantly influenced by the physicochemical properties of AgNP, including size, shape, agglomeration, coatings, and surface charge, which in turn affects the toxic effects (Akter et al. 2018; Kim and Ryu 2013). After cellular internalisation, AgNP exert a range of harmful effects, including oxidative stress (OS), inflammation, DNA damage, cell cycle disruption, and ultimately, cell death (Dos Santos et al. 2014; Kim and Ryu 2013; Nie et al. 2023; Sousa et al. 2022). Therefore, it is imperative to investigate these mechanisms to gain a comprehensive understanding of their actual impact on human health and to identify strategies for mitigating adverse effects.

Flavonoids, polyphenolic compounds with a 15-carbon skeleton, are common in the daily diet and beverages like tea and wine. Considering that these compounds exhibit a multitude of beneficial biological activities, such as anti-inflammatory and antioxidant activities (Chen et al. 2023), flavonoids hold promise for protecting against AgNP-induced toxicity.

The present review of the literature aims to provide a comprehensive overview of the potential protective effects of flavonoids against AgNP-induced toxicity. This literature review will begin by exploring the main mechanisms of AgNP-induced toxicity, followed by a summary of the biological relevance of flavonoids. Finally, an analysis will be presented of the existing research on the flavonoids protective effects against AgNP-induced toxicity. This section include information of the effects of flavonoids when applied separately from AgNP; the effects of modified-AgNP with flavonoids (e.g., as coatings, core–shell or loaded); and, a brief overview of the synergistic interactions of AgNP and flavonoids in modulating biological properties.

## Mechanisms of silver nanoparticles induced toxicity

**Fig. 1:**
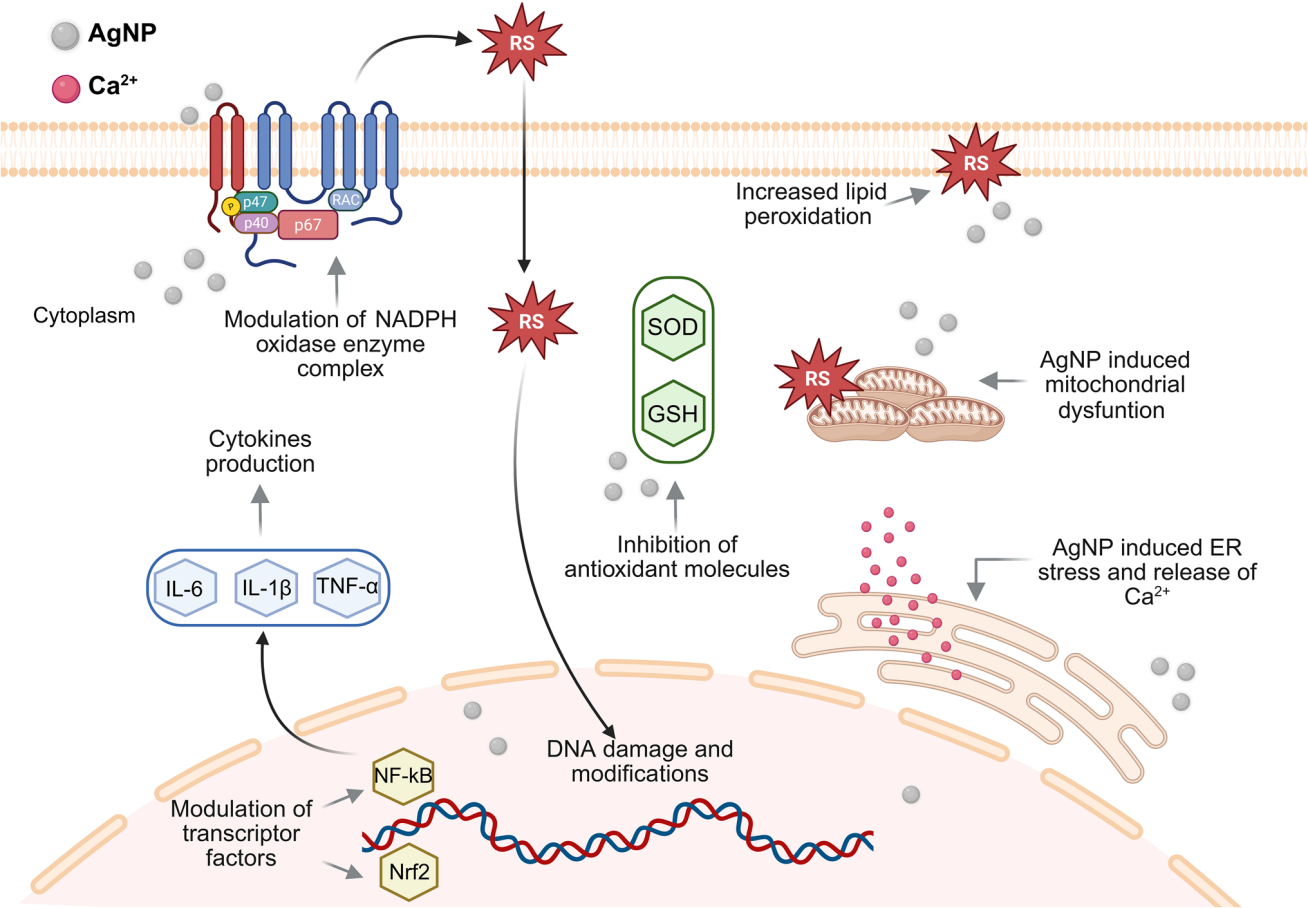
Mechanisms of AgNP toxicity.* AgNP* Silver nanoparticles, *ER* Endoplasmic reticulum,* GSH* Glutathione, * IL-1β* Interleukin 1β,* IL-6* Interleukin 6,* NADH* Nicotinamide adenine dinucleotide,* NF-kB* Nuclear factor kappa beta,* Nrf2* Nuclear factor erythroid 2-related factor 2, RS reactive species,* SOD* Superoxide dismutase,* TNF α* Tumour necrosis factor-alpha. Figures created with Biorender image bank

The mechanisms of AgNP-induced toxicity are already elegantly reviewed for example by Sousa et al. (Sousa et al. 2022), Zhang et al. (Zhang et al. 2014), Nie et al. (Nie et al. 2023), or Kim et al. (Kim and Ryu 2013). The literature suggests that AgNP-induced toxicity arises from multiple mechanisms (Fig. 1), which will be briefly described in this section. These mechanisms include OS, pro-inflammatory response, mitochondria dysfunction, endoplasmic reticulum (ER) stress, DNA damage, and cell death.

One primary mechanism by which AgNP induces cellular damage involves the disruption of the balance between the generation and neutralisation (via antioxidant defences) of reactive species (RS), which damages cells and tissues (Pizzino et al. 2017). AgNP induce the production of RS through the activation of nicotinamide adenine dinucleotide phosphate hydrogen (NADPH) oxidase enzyme complex, namely by increasing the production of superoxide anion radicals (O_2_^•−^) and consequently other RS (Freitas et al. 2020; Sousa et al. 2022). Within the body, AgNP are oxidised by O_2_ and/or other molecules resulting in the production of Ag^+^, which form stable bonds with sulphur containing biological molecules (e.g., cysteine). Some of these sulphur containing molecules are also antioxidant molecules, as glutathione (GSH) and superoxide dismutase (SOD), thereby leading to loss of the effectiveness of antioxidant defence mechanisms (Buglak et al. 2019; Nie et al. 2023; Sousa et al. 2022), leading to OS and potentially irreversible damage to cellular proteins and structures, culminating in cell death (Dos Santos et al. 2014; Grzelak et al. 2018). In addition to the cellular mechanisms, evidence suggests that AgNP contribute to OS through acellular pathways (Zhang et al. 2014). It has been demonstrated that AgNP can directly generate RS via surface redox reactions, particularly through Fenton-like reactions in the presence of hydrogen peroxide (H_2_O_2_) and acidic environments (Zhang et al. 2014). Furthermore, the presence of H_2_O_2_ within a cell, even at low doses, has been demonstrated to accelerate the dissolution of AgNP. This, in turn, increases Ag^+^ availability, which leads to the production of O_2_^•−^ and other reactive oxygen species (ROS) through subsequent interactions with O_2_ (Kim and Ryu 2013).

Another mechanism by which AgNP exposure may induce cytotoxic effects is through the modulation of pro-inflammatory mediators, including transcription factors and inflammatory cytokines (Choudhary et al. 2022; Sousa et al. 2022). AgNP prompt pro-inflammatory responses in immune cells (e.g., neutrophils and monocytes) and endothelial cells (e.g., umbilical vein endothelial and cerebral microvascular endothelial cells) (Ninan et al. 2020; Shi et al. 2014; Sokołowska et al. 2017). The immune cells recognise AgNP as foreign stimuli, thereby initiating a cascade of inflammatory reactions (Ninan et al. 2020; Sousa et al. 2022). This result in the activation of different inflammatory signalling pathways including the activation of nuclear factor kappa beta (NF-kB) and mitogen-activated protein kinase (MAPK) pathways. However, other subfamilies of MAPK, namely extracellular signal-regulated kinases [e.g. C-jun N-terminal kinase (JNK)], are also activated by AgNP (Ninan et al. 2020; Sousa et al. 2022). These pathways modulate the transcription of genes, involved in the inflammatory response, leading to the expression of pro-inflammatory mediators, namely pro-inflammatory cytokines as interleukin (IL)−8, tumour necrosis factor-α (TNF-α), IL-1β, and IL-6. Additionally, AgNP may also interfere with the nuclear factor erythroid 2-related factor 2 (Nrf2), a regulator of cellular redox balance that influences cytoprotective agents like (HO-1) (Noga et al. 2023; Sousa et al. 2022).

The presence of AgNP in mitochondria has already been documented, and there is evidence suggesting that this accumulation may result in mitochondrial dysfunction (Nie et al. 2023; Sousa et al. 2022). Once inside cells, AgNP can directly interact with mitochondria, disrupting the mitochondrial membrane potential and impairing the respiratory chain. This disruption results in the overproduction of RS, which further inhibits the production of adenosine triphosphate (ATP) (McShan et al. 2014; Sousa et al. 2022), a molecule essential for various vital cellular functions, as muscular contraction, protein cofactor, or in signalling of key bioprocesses (Barclay 2015; Chu et al. 2022). It is noteworthy that AgNP can penetrate the inner mitochondrial membrane, leading to damages not induced by RS (Akter et al. 2018; Li et al. 2020). This results in the mitochondrial swelling and damage to the mitochondrial cristae structure, as well as influencing mitochondrial fusion and fission. Defects in these processes also impact mitochondrial function, leading to a decrease in the ATP production (Li et al. 2020; Zhang et al. 2022b). AgNP also trigger mitochondrial membrane leakage through activation of tumour protein p53, leading to upregulation of apoptotic inducers like B-cell leukaemia 2 protein (Bcl-2)-associated X protein (Bax) or Bcl-2 homologous antagonist killer. Concurrently with this upregulation, a downregulation of anti-apoptotic mediators, such as Bcl-2, also occurs (Kim and Ryu 2013; Sousa et al. 2022).

Additionally, it is also described that AgNP induce damage to the ER, which plays an essential role in protein folding, calcium storage and regulation, and the transfer of redox signals to the cytoplasm (Nie et al. 2023; Zhang et al. 2022b). AgNP have been demonstrated to induce alterations in stress sensing proteins of the ER, including protein kinase R—like ER kinase (PERK), which ultimately results in ER homeostasis disruption and subsequent damage (Chen et al. 2016). Moreover, AgNP have been observed to extend the length of the ER–mitochondria contact site, thereby promoting an increase in ER proteins and the mitochondria-associated membranes. AgNP can also modify the function of inositol-3-phosphate receptor and, as a consequence, a transfer of Ca^2+^ occurs from the ER to the mitochondria, promoting an overload of mitochondrial Ca^2+^, ultimately triggering apoptosis (Zhang et al. 2022b). Additionally, AgNP disrupt Ca^2+^ homeostasis, impacting protein regulation processes such as the calnexin/calreticulin cycle (Zhang et al. 2022b).

The cellular uptake of AgNP has been demonstrated to increase the interaction with DNA, leading to structural and functional changes and disrupting repair pathways (Nallanthighal et al. 2017; Rodriguez-Garraus et al. 2020). AgNP may alter DNA directly and indirectly via OS, destabilising cellular functions. Direct interactions with DNA can hinder replication and transcription (Encinas-Gimenez et al. 2024). Regarding to the AgNP indirect effects, the AgNP-induced RS cause oxidative damage to the DNA, thereby inducing pro-mutagenic lesion through the formation of 8-oxo-7,8-dihydro-2-deoxyguanine (8-oxoG), and downregulates 8-oxoguanine DNA glycosylase 1 (OGG1), a repair enzyme that removes oxidised guanine (8-oxoG), leading to genotoxicity (Mytych et al. 2017; Nallanthighal et al. 2017). AgNP may also influence the induction of DNA hypermethylation as a downstream effect of the activation of the p53 or p21 activation, promoting cells’ apoptosis (Akter et al. 2018; Gurunathan et al. 2018). It has also been found that it is also possible for AgNP to cause incorrect segregation of chromosomes during mitosis, causing abnormal division and lead to epigenetic deregulation, which may exert long-term effects on reprogramming gene expression (Akter et al. 2018; Garcia et al. 2019). Other AgNP-induced damages that have being already reported are DNA deletions, micronuclei formation, irreversible chromosomal damage, and double-strand breaks (Encinas-Gimenez et al. 2024; Garcia et al. 2019; Kim and Ryu 2013; Nallanthighal et al. 2017). These genetic alterations can lead to severe cellular dysfunction, compromising genomic stability and potentially triggering carcinogenesis or other long-term adverse effects (Encinas-Gimenez et al. 2024; Garcia et al. 2019; Kim and Ryu 2013; Nallanthighal et al. 2017).

The AgNP-induced DNA damage/mutations can consequently result in a cell cycle deregulation, inhibition of the proliferation, or even cell death (Kim and Ryu 2013). The inhibition of proliferation occurs when cells become arrested in one or more phases of the cell cycle. Cell growth can be interrupted in the G1, S, or G2/M phase, with the specific phase of interruption varying depending on the cell type (Garcia et al. 2019; Kim and Ryu 2013; Zhang et al. 2014).

The mentioned adverse effects of AgNP can vary with their physicochemical characteristics, including their size, shape, agglomeration state, and surface charge or coating agents (Akter et al. 2018).

Regarding the size, several studies have reported that smaller-sized AgNP induce more toxicity (Kim et al. 2012; Kim and Ryu 2013). In fact, smaller AgNP have a greater surface area-to-volume ratio and, consequently, can be easily internalised in cells, ultimately enhancing their toxicity by increasing the release of Ag^+^ ions and their reactivity with cellular components (Ferdous and Nemmar 2020; Kim and Ryu 2013). The diverse shapes that AgNP can assume also influence the degree of toxicity. Different shapes can result in variations in physicochemical properties such as specific surface area or surface charge, resulting in distinct interactions with cellular components (Akter et al. 2018). To illustrate, spherical AgNP can undergo endocytosis faster, whilst non-spherical NP may result in increased blood circulation time and subsequent elevation of toxicity (Panariti et al. 2012). AgNP with irregular surfaces can promote interactions with cellular sites, thereby promoting toxic effects (Nie et al. 2023). The utilisation of coatings has been demonstrated to enhance the stability, biocompatibility, and other properties of materials. However, the specific type of coating (organic or inorganic) can influence the level of toxicity induced in cells (Kim and Ryu 2013). For example, AgNP coated with polysaccharide showed less toxicity than AgNP coated with chitosan, in the same way that AgNP coated with carbohydrates are less toxic than uncoated ones (Akter et al. 2018; Vukoje et al. 2022). The coating can also modify the charge of the particles, which subsequently influences their toxicity within cells. For instance, positively charged NP are regarded as more effective drug delivery vehicles for anticancer drugs, given their capacity to persist in the bloodstream for extended periods relative to their negatively charged counterparts (Ferdous and Nemmar 2020). This property leads to stronger electrostatic interactions with cells, thereby facilitating endocytic uptake. This is due to an attractive force between the positively charged AgNP and the negative charge of the cell membranes (Ferdous and Nemmar 2020). Concerning the state of agglomeration or aggregation, this can influence the manner in which the AgNP interact with cells. This may result in a reduction in cellular binding and uptake, accompanied by an increase in instability and toxicity. Furthermore, this effect is not a straightforward consequence of particle size; rather, it is a complex interplay between particle and cell properties (Lankoff et al. 2012).

The extent of cytotoxicity caused by AgNP is also contingent upon a number of factors, including AgNP concentration, the type of cells being exposed, and the duration of exposure. These dependencies highlight the importance of considering these variables when assessing the potential harm of AgNP (Akter et al. 2018; Nie et al. 2023). It is essential to ascertain the minimum concentration of AgNP that induces toxicity. Furthermore, long-term exposure to AgNP also results in a more pronounced toxic effect than that observed following short periods of exposure to the NP (Akter et al. 2018).

## Flavonoids

**Fig. 2:**
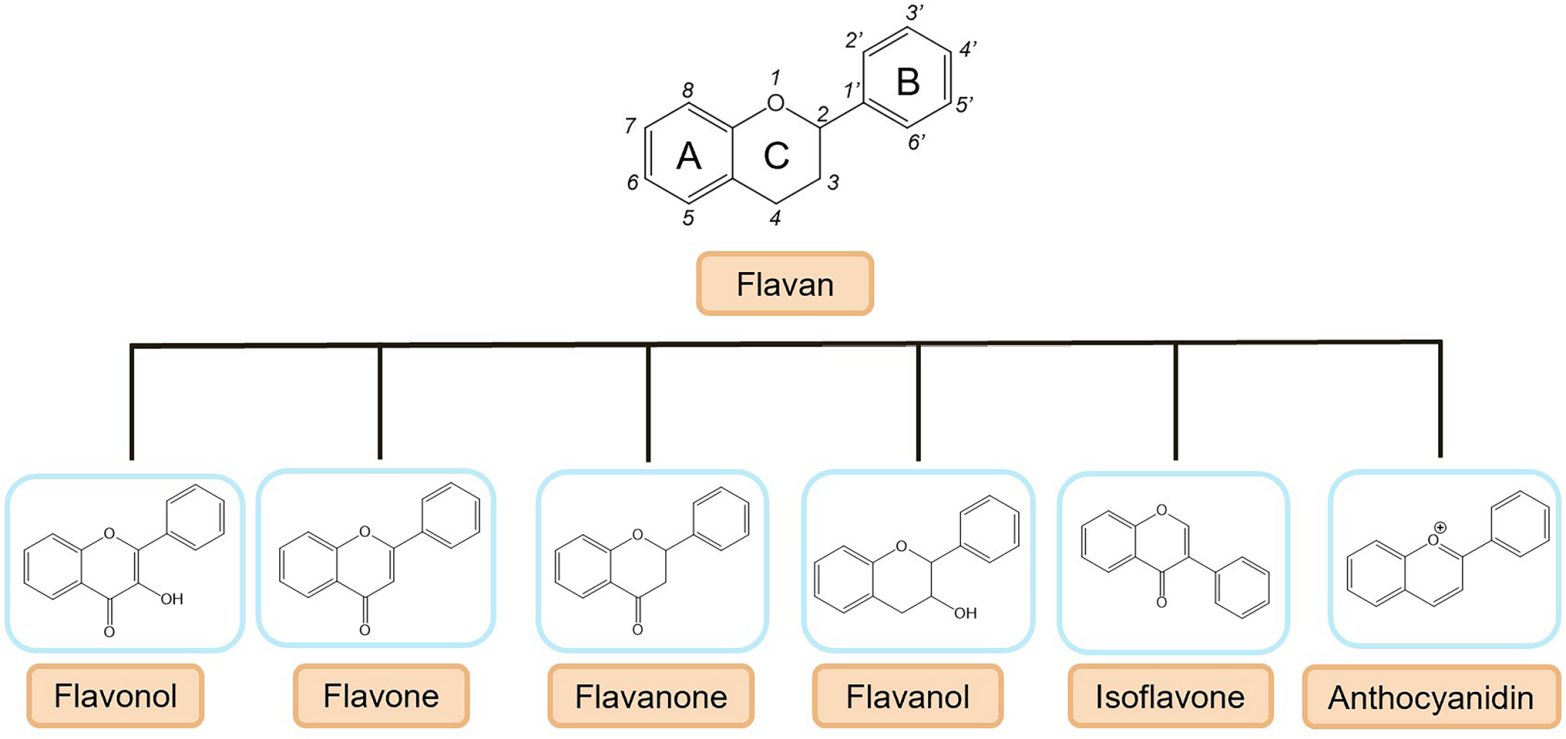
Basic structure of flavonoids and subclasses

The discovery of flavonoids dates back to the 1930’s, when a compound extracted from lemon juice was found to reduce capillary permeability and bleeding in scurvy-afflicted guinea pigs, a condition where vitamin C alone proved ineffective (Akhlaghi and Foshati 2017). This led to the nomination of flavonoids as vitamin P, although this terminology was later dismissed (Akhlaghi and Foshati 2017). Since then, the quest for this advantageous phytochemical has intensified, with over 10.000 flavonoids already identified and isolated (Ullah et al. 2020). These compounds are natural secondary metabolites found in a wide range of fruits, vegetables, herbs, and other botanical sources. They belong to a group of polyphenolic compounds characterized by a 15-carbon skeleton consisting of two benzene rings (A and B as shown in Fig. 2) linked by a heterocyclic ring (C) (Dias et al. 2021; Ullah et al. 2020). Furthermore, flavonoids can be found in two different forms: the free form (aglycones) and the glycosylated form (glycones) (Dias et al. 2021; Ullah et al. 2020).

Flavonoids are classified according to their structural characteristics, degree of unsaturation, and oxidation state of the carbon ring, resulting in six distinct subclasses (Chen et al. 2023; Kumar and Pandey 2013). The subclasses differ in the substitution pattern of the C ring and the degree of oxidation. Additionally, individual flavonoid compounds within each subclass differ in their A and B rings. These subclasses include flavones, flavanones, flavanols, flavonols, isoflavones, and anthocyanidins (Fig. 2) (Liga et al. 2023).

### Biological activities of flavonoids

Flavonoids exhibit a wide range of biological properties, as highlighted in several elegantly conducted studies, including antioxidant (Shen et al. 2022), anti-inflammatory (Al-Khayri et al. 2022), anticancer (Farhan et al. 2023), antimicrobial (Cushnie and Lamb 2005; Badshah et al. 2021), antidiabetic (Al-Ishaq et al. 2019), hepatoprotective activity (Gajender et al. 2023), osteogenic properties (Sekaran et al. 2022), cardioprotective (Chen et al. 2023; Sánchez et al. 2019), and neuroprotective (Calis et al. 2020) (Table 1). However, the biological properties of these compounds are contingent upon a variety of factors, including their structural characteristics and the presence of functional groups (Dias et al. 2021).

**Table 1:** Biological activities of flavonoids

Activity	Action	Flavonoids	References
Antioxidant	– Suppression of RS formation; – Scavenging RS; – Activation of metal-chelating activity – Upregulation or protection of antioxidant defences (e.g. SOD);	Quercetin, naringenin, luteolin, hesperitin, daidzein, eriodictyol, silymarin, chrysin, acacetin, diosmetin, genistein, glycitein, formononetin, morin, kaempherol, myricetin, pelargonidin, petunidin	(Kumar and Pandey 2013; Liga et al. 2023; Shen et al. 2022; Ullah et al. 2020)
Anti-inflammatory	– Inhibition of inflammatory pathways; – Reduction of the production of pro-inflammatory mediators, e.g. (cytokines)	Quercetin, naringenin, hesperitin, eriodictyol, silymarin, rutin, luteolin, chrysin, acacetin, diosmetin, genistein, formononetin, morin, kaempherol, myricetin, catechin, epigallocatechin, afzelechin, cyanidin, delphinidin, pelargonidin	(Al-Khayri et al. 2022; Ferraz et al. 2020; Liga et al. 2023)
Anticancer	– Induction of cell death; – Inhibition the cellular growth; – Inhibition angiogenesis;	Chrysin, luteolin, naringenin, hesperitin, eriodictyol, silymarin, acacetin, diosmetin, genistein, glycitein, formononetin, morin, catechin, epigallocatechin, afzelechin	(Farhan et al. 2023; Kumar and Pandey 2013; Liga et al. 2023; Ullah et al. 2020)
Antimicrobial	– Inhibition of bacteria and fungal growth; – Inhibition of nucleic acid synthesis; – Inhibition of energy metabolism; – Inhibition the efflux-mediated pumping systems; – Inhibition the multiplication of virus;	Baicalein, hesperitin, genistein, daidzein, glycitein, formononetin, quercetin, kaempherol, rutin, catechin, epigallocatechin, afzelechin, malvidin	(Chen et al. 2023; Liga et al. 2023; Ullah et al. 2020; Badshah et al. 2021; Liga et al. 2023; Ninfali et al. 2020)
Antidiabetic	– Enhancement of β-cell proliferation; – Promotion of insulin secretion and decrease of resistance; – Regulation of glucose metabolism in the liver; – Modulation the activity of enzymes directly related with the pathophysiology of diabetes;	Hesperitin, acacetin, morin, myricetin, malvidin	(Al-Ishaq et al. 2019; Liga et al. 2023; Proença et al. 2022; Rocha et al. 2022; Rocha et al. 2019; Ullah et al. 2020)
Hepatoprotective	– Protection against hepatic tissue damage; – Protection of liver cells against inflammation and OS;	Naringenin, eriodictyol, myricetin, malvidin	(Casas-Grajales and Muriel 2015; Kumar and Pandey 2013; Liga et al. 2023; Tapas et al. 2008)
Osteogenic properties	– Enhancement of osteoblastogenesis; – Inhibition of osteoclastogenesis;	Naringenin, genistein, daidzein	(Liga et al. 2023; Sekaran et al. 2022; Zhang et al. 2022a)
Cardioprotective	– Decreased blood pressure; – Antiplatelet aggregation; – Reduction of the content of triglycerides, total cholesterol, and low-density lipoprotein cholesterol; – Regulation endothelial cell apoptosis;	Chrysin, hesperitin, acacetin, morin, myricetin, catechin, epigallocatechin, afzelechin	(Chen et al. 2023; Liga et al. 2023; Sánchez et al. 2019)
Neuroprotective	– Improvement of cognitive function; – Suppression of neuroinflammation;	Apigenin, baicalein, wogonin, chrysin, acacetin, formononetin, myricetin	(Basli et al. 2012; Calis et al. 2020; Liga et al. 2023)

*OS* Oxidative stress, *RS* Reactive species, *SOD* Superoxide dismutase

### Absorption, bioavailability, and metabolism of flavonoids

One of the main concerns regarding the use of flavonoids pertains to their chemical and biophysical properties, including low solubility, limited bioavailability, and pharmacokinetics (Liga et al. 2023). Typically, only 5–10% of ingested flavonoids are absorbed in the small intestine, and bioavailability can vary considerably across different flavonoid subclasses and individual compounds within each subclass (Thilakarathna and Rupasinghe 2013; Yang et al. 2023). The majority of food flavonoids are present in glycoside forms, conjugated with sugars (Akhlaghi and Foshati 2017), although aglycones forms are occasionally found, and the flavanols, structurally, rarely occurs as glycosides (Akhlaghi and Foshati 2017; Chen et al. 2023; Luo et al. 2022). Therefore, the flavonoids glycosides, due to their hydrophilic nature, exhibit limited permeability across cellular membranes. Consequently, the absorption of these compounds requires either the hydrolysis of sugar conjugates or the involvement of a specific active transport mechanism in the intestine, such as active sodium-dependent glucose transporter (SGLT1) (Ribeiro et al. 2018).

**Fig. 3:**
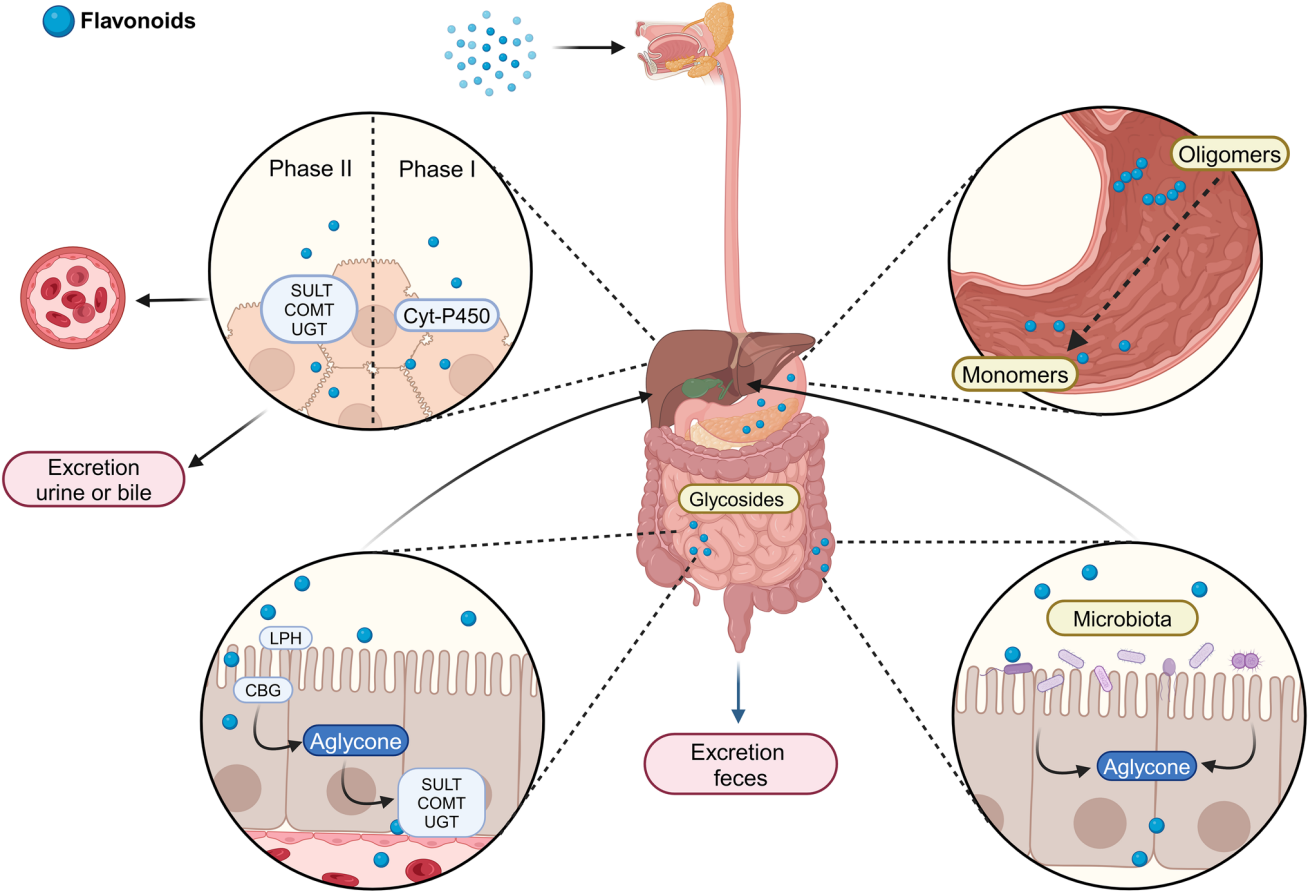
Representative figure of flavonoid metabolism.* CBG* Cystolic β-glucosidase, * COMT* Catechol-O-methyltransferase,* Cyt-P450* Cytochrome P450,* LPH* Lactase phlorizin hydrolase,* SULT* Sulfotransferases,* UGT* UDP-glucuronosyltransferases. Figures created with Biorender image bank

Once ingested, flavonoids (oligomers) reach the stomach, where the acidic environment aids their conversion into simpler monomeric forms (Al-Ishaq et al. 2019; Kumar and Pandey 2013). Subsequently, the flavonoids, in the form of glycosides, reach the small intestine, undergoing further transformations, including hydrolysis by lactase-phlorizin-hydrolase (LPH) or cystolic β-glucosidase (CBG) (Kamiloglu et al. 2021). This process generates aglycones, which, subsequently, can then participate in conjugation forming metabolites like glucuronides, sulphates, and methylated derivatives—smaller phenolic compounds that can enter the bloodstream (Akhlaghi and Foshati 2017; Al-Ishaq et al. 2019; Ribeiro et al. 2018). Following these biotransformation, the flavonoids may be absorbed or, alternatively, remain unabsorbed in the small intestine, reaching posteriorly, the colon (Fig. 3) (Al-Ishaq et al. 2019; Ribeiro et al. 2018).

Once absorbed in the small intestine, flavonoids are transported to the liver, where they undergo further transformations, namely, phase I/II metabolism (Al-Ishaq et al. 2019; Alsawaf et al. 2022; Ribeiro et al. 2018). Phase I involves oxidation, mediated by cytochrome P450 enzymes whilst phase II includes further conjugation reactions, such as methylation, sulfation, and glucuronidation—reactions similar to those occurring in the small intestine. These conjugated derivatives are eventually excreted via bile and urine or continue to circulate in the bloodstream (Al-Ishaq et al. 2019; Alsawaf et al. 2022; Ribeiro et al. 2018).

In the case of unabsorbed flavonoid in form of glycosides in the small intestine, structural modifications occur in the colon. These alterations are subsequently enacted as a result of the action of the colonic microbiota acids (Murota et al. 2018; Thilakarathna and Rupasinghe 2013). The intestinal microbiota plays a pivotal role in the hydrolysis of glycosides and the degradation of released flavonoid aglycones into phenolic acids (Murota et al. 2018; Thilakarathna and Rupasinghe 2013). These transformations enable further excretion of the compounds (Ribeiro et al. 2018; Thilakarathna and Rupasinghe 2013).

## Flavonoids as promising protective agents against the adverse effects induced by silver nanoparticles

### Methodology of search

**Fig. 4:**
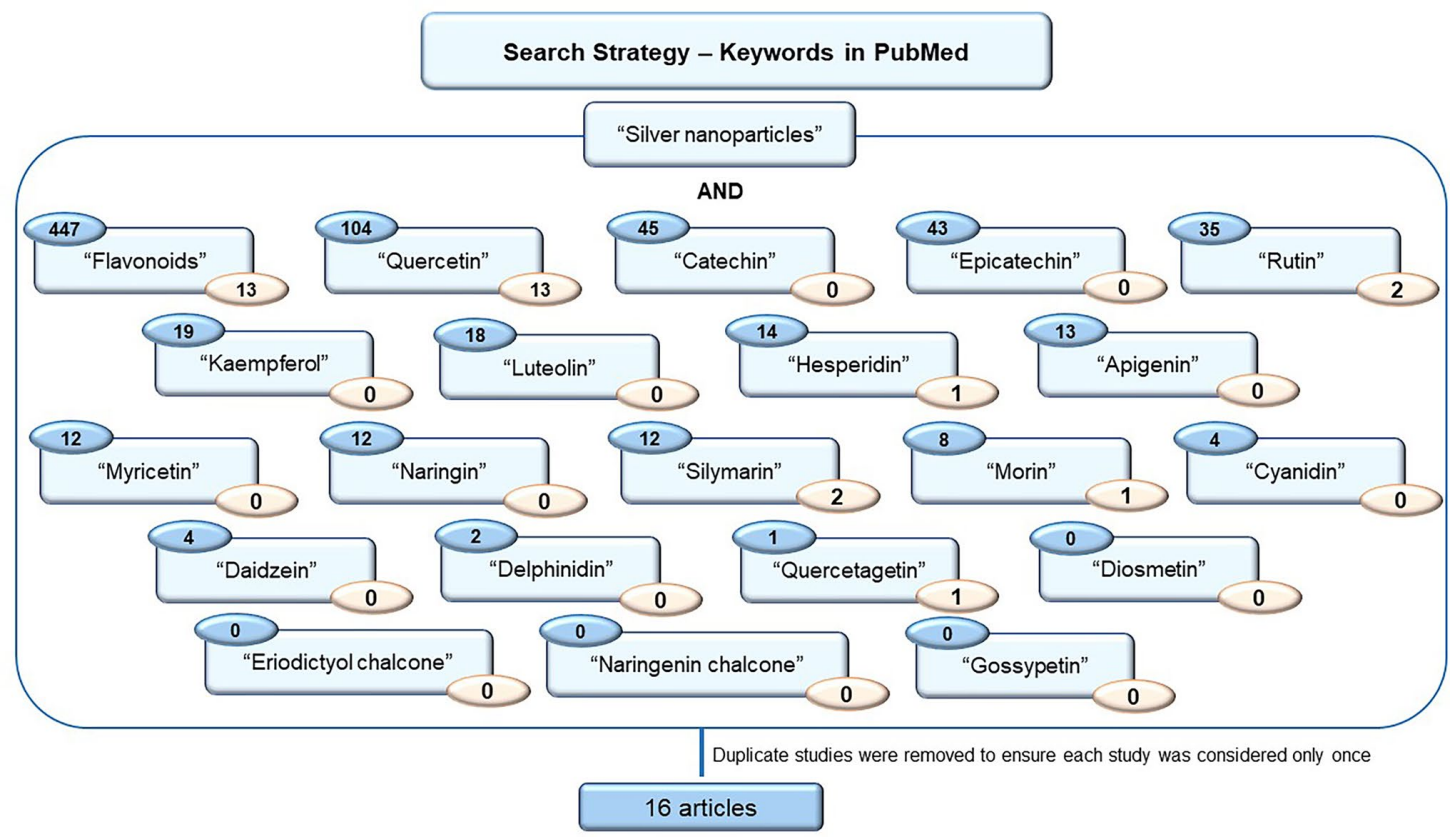
Diagram of articles search on PubMed databased, until August 2024. Blue circles—number of articles retrieved; orange circle: number of articles included

A literature review was conducted on the potential protective effects of flavonoids against the adverse impacts of AgNP, using the PubMed database up to August 2024. The search employed the keywords “silver nanoparticles” or “Ag nanoparticles” and “flavonoids” (447 articles) or the most common flavonoids “quercetin “ (104 articles), “catechin” (45 articles), “epicatechin” (43 articles), “rutin” (35 articles), “kaempferol” (19 articles), “luteolin” (18 articles), “hesperidin” (14 articles), “apigenin” (13 articles), “myricetin” (12 articles), “naringin” (12 articles), “silymarin” (12 articles), “morin” (8 articles), “cyanidin” (4 articles), “daidzein” (none), “delphinidin” (2 articles), “quercetagetin” (1 article), “naringenin chalcone” (none), “eriodictyol chalcone” (none), “gossypetin” (none), and “diosmetin” (none) (Fig. 4).

Considering the literature research carried out, this review is divided into two sections, based on the following considerations:

- The protective effects of flavonoids against AgNP-induced toxicity, with flavonoids introduced separately from AgNP—thirteen articles.
- The protective effects of flavonoids against AgNP-induced toxicity when present in modified-AgNP, including as coatings, core–shell structures, or in loaded forms—three articles.

To ensure the discussion remains focused on the direct effects of flavonoids, studies using pure flavonoids were selected, thereby avoiding the potential variability introduced by plant extracts or other compounds. As the majority of the articles identified in the preliminary search specifically addressed the green synthesis of AgNP (involving the use of flavonoids in the process of synthesis), these studies were excluded as they did not evaluate the protective effects of flavonoids themselves. Furthermore, studies investigating the protective properties of flavonoids in modified-AgNP, and only those that compared the effects of flavonoid modified-AgNP and unmodified-AgNP were selected. Additionally, although studies exploring the synergistic effects between AgNP and flavonoids were not included amongst the sixteen articles selected for the two primary topics, they were incorporated into the final section of the review. Only articles written in English were included in this review.

### Protective effects of flavonoids against AgNP-induced toxicity when administered separately

Table 2 provides a summary of both in vitro (six articles) and in vivo (seven articles) studies demonstrating the potential protective effects of flavonoids against the adverse impacts of AgNP, when administered separately. Amongst the flavonoids, quercetin was identified as the most commonly utilised in the thirteen articles analysed, featuring in seven of these studies (Elblehi et al. 2022; Goodarzi et al. 2023; Martirosyan et al. 2014; Martirosyan et al. 2016; Rufino et al. 2021; Seyedi et al. 2021; Sirotkin et al. 2021). The remaining studies used morin (two articles) (Arisha et al. 2019; Rufino et al. 2021), silymarin (two articles) (Faedmaleki et al. 2016; Veisi et al. 2021), and rutin (two articles) (Ahmed and Hussein 2017; Essawy et al. 2021). The less-studied flavonoids, with only one study in the literature include quercetagetin, luteolin, myricetin, gossypetin, diosmetin (Rufino et al. 2021), kaempferol (Martirosyan et al. 2014), hesperidin, and naringin (Ali et al. 2022). Other interesting fact is that most of the flavonoids tested are flavonols (seven of the twelve flavonoids), flavanones (three of the twelve flavonoids), and flavones (two of the twelve flavonoids). This fact demonstrates that flavonols are preferentially selected to study the protective effects against AgNP. This may be related to the fact that flavonols, which are primary flavonoids in nature, are found in various foods in our diet, such as apples, berries, grapes, tomatoes, and onions (Chagas et al. 2022; Chen et al. 2023). Some of the compounds of this subclass, such as quercetin, are the one of the most extensively study flavonoid (Aghababaei and Hadidi 2023; Carrillo-Martinez et al. 2024) and they demonstrate pronounced biological effects, including antioxidant and anti-inflammatory effects (Chagas et al. 2022).

**Table 2:**
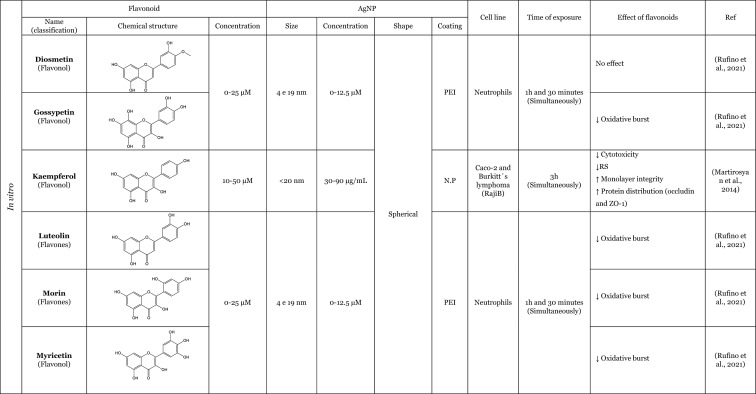

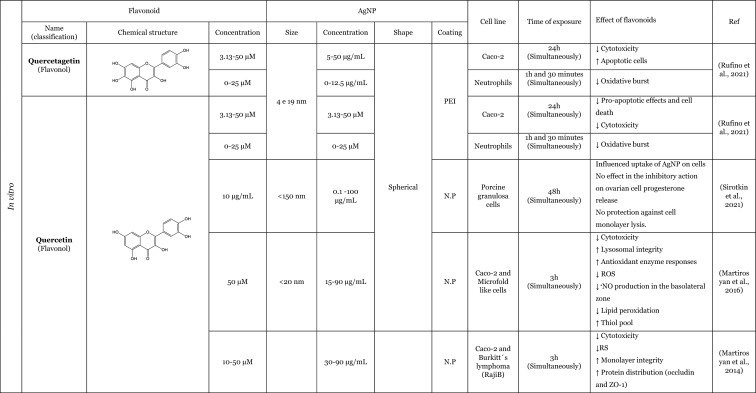

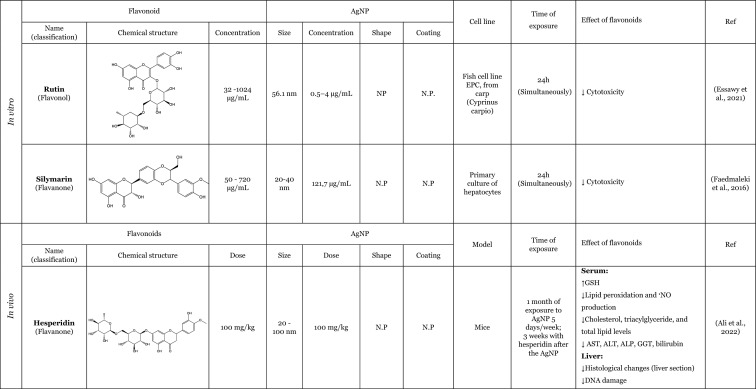

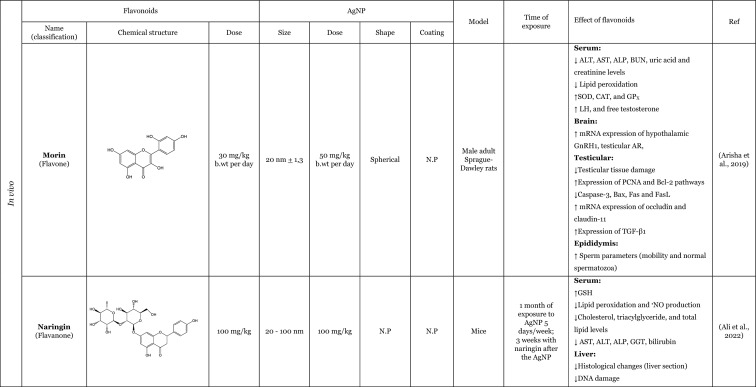

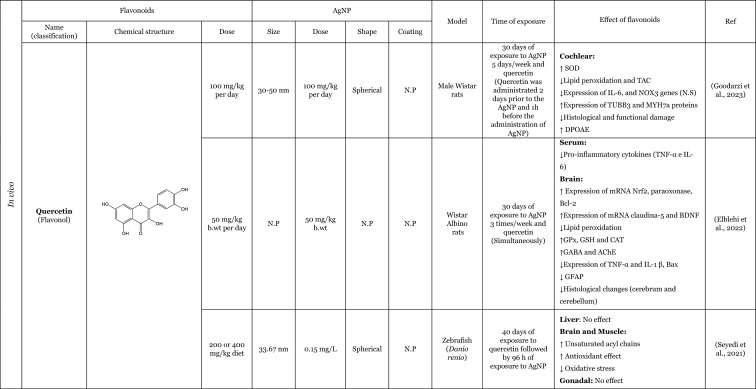

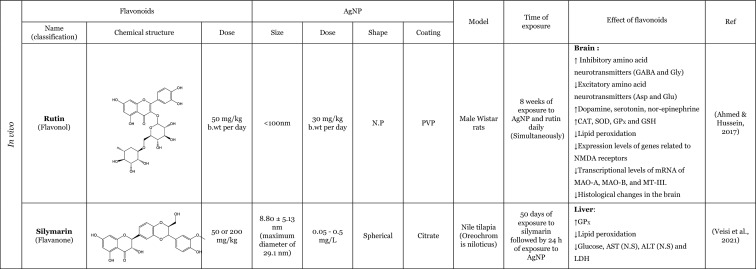
Overview of the in vitro and in vivo studies exploring the potential protective effects of flavonoids against toxic effect induced by AgNP

**↓** Decrease, **↑** Increase, *AChE* Acetylcholinesterase, *ALP* Alkaline phosphatase, *ALT* Alanine aminotransferase, *AST* Aspartate aminotransferase, *Bax* Bcl-2-associated X protein, *Bcl*−2 B-cell lymphoma 2, *BDNF* Brain-derived neurotrophic factor, *BTB* blood–testis barrier, *BUN* Blood urea nitrogen, *CAT* Catalase, *DNA* Deoxyribonucleic acid, *DPOAE* Distortion product otoacoustic emission, *GABA* Gamma-aminobutyric acid, *GFAP* Glial fibrillary acidic protein, *GGT* Gamma-glutamyltransferase, *GPx* Glutathione peroxidase, *GSH* Glutathione, *LDH* lactase dehydrogenase, *IL-1β* Interleukin 1β, *IL-6* Interleukin 6, *LH* Luteinizing hormone, *MAO-A* Monoamine oxidase A, *MAO-B* Monoamine oxidase B, *mRNA* Messenger ribonucleic acid, *MT-III* Metallothionein III, *MYH7a* Myosin Heavy Chain 7, *NF*-*кB* Nuclear factor kappa B, *NMDA* N-methyl-D-aspartate, ^•^*NO* Nitric oxide, *NOX3* NADPH oxidase 3, *Nrf2* Nuclear factor erythroid 2-related factor 2, *N*.*P*. Information not provided, *N*.*S*. Not significant, *PCNA* Proliferating cell nuclear antigen, *PVP* Polyvinylpyrrolidone, *PEI* Polyethylenimine, *ROS* Reactive oxygen species, *RS* Reactive species, *SOD* Superoxide dismutase, *TAC* Total antioxidant capacity, *TGF*-*β* Transforming growth factor beta, *TNF*-*α* Tumour necrosis factor α, *TUBB*−3 β-tubulin III, *ZO*-*1* Zonula occludens-1

Seven studies were conducted in vivo, which is of great importance for providing a physiological approximation of the protective effects of flavonoids. A total of five studies used rats or mice as experimental subjects (Ahmed and Hussein 2017; Arisha et al. 2019; Elblehi et al. 2022; Goodarzi et al. 2023; Seyedi et al. 2021), whilst two studies utilised fish models (Seyedi et al. 2021; Veisi et al. 2021). In consideration of the in vitro models, which represent six of the thirteen studies included in this review, it is notable that several of these employ cellular monoculture models, such as the Caco-2 cell line (three articles) (Martirosyan et al. 2014; Martirosyan et al. 2016; Rufino et al. 2021), neutrophils (one article) (Rufino et al. 2021), porcine granulosa cells (one article) (Sirotkin et al. 2021), primary hepatocytes (one article) (Faedmaleki et al. 2016), fish cell line EPC (one article) (Essawy et al. 2021), and cellular co-culture models (two studies), using Caco-2 and RajiB cells (Martirosyan et al. 2014) as well as Caco-2 and Microfold like cells (Martirosyan et al. 2016). In the majority of the studies, the incubations were performed using spherical AgNP. The application of flavonoids was typically conducted concurrently with the administration of AgNP, as evidenced by nine articles in the literature. However, in certain instances, flavonoids were only introduced subsequent to the exposure to AgNP (Ali et al. 2022) or either before exposure to AgNP (Seyedi et al. 2021; Veisi et al. 2021). Interestingly, in one study, the flavonoid was administered independently, for a period of 2 days prior to the AgNP, and for 1 h before the AgNP for a total of 30 days (Goodarzi et al. 2023).

The in vitro and in vivo studies explored the protective effects of flavonoids applied separately, on the production of RS and lipid peroxidation induced by AgNP. The findings indicate that flavonoids have the ability to decrease RS levels, which, in turn, results in a decline in lipid peroxidation. The observed effects exhibited that flavonoids effectively contribute to the maintenance of cellular redox balance and to the protection of cellular components (Ahmed and Hussein 2017; Ali et al. 2022; Arisha et al. 2019; Elblehi et al. 2022; Essawy et al. 2021; Faedmaleki et al. 2016; Goodarzi et al. 2023; Martirosyan et al. 2014, 2016; Rufino et al. 2021; Seyedi et al. 2021; Veisi et al. 2021). For instance, an in vitro study conducted by Martirosyan et al. (2014) using Caco-2 cells exposed simultaneously to 30–90 μg/mL of < 20 nm-sized AgNP and quercetin and kaempferol, at concentrations of 10–50 µM, showed the potential of the flavonoids in reducing RS. In other study carried out by the same authors (Martirosyan et al. 2016) denoted that in monoculture using Caco-2 cells exposed simultaneously to 15–90 µg/mL of < 20 nm-sized AgNP and quercetin at a concentration of 50 µM. The study demonstrated that quercetin was able to reduce RS production. It is noteworthy that, with regard to nitric oxide (^•^NO) levels, quercetin exhibited intriguing effects. In the co-culture system, an elevation of ^•^NO was observed in the apical zone, where Caco-2 cells were present. Conversely, in the basolateral zone, where microfold-like cells were applied, a slight reduction in ^•^NO levels was observed (Martirosyan et al. 2016). Rufino et al. (2021), using Caco-2 cells, investigated the impact of a panel of flavonoids (quercetin, quercetagetin, diosmetin, luteolin, morin, myricetin, and gossypetin) when simultaneously exposed to 5 to 50 µg/mL AgNP coated with polyethyleneimine (PEI-AgNP) in two sizes (4 and 19 nm). It was observed that both sizes of PEI-AgNP induced the production of ^•^NO. However, none of the flavonoids were able to reduce the production of ^•^NO. In this same study, it was also demonstrated that PEI-AgNP, at a range of concentrations between 0 and 12.5 µM, were able to induce the neutrophils oxidative burst. Quercetagetin and quercetin were the most effective flavonoids against the neutrophils oxidative burst induced by AgNP of 4 nm. However, in the case of 19 nm AgNP, luteolin and morin emerged as the most effective flavonoids (Rufino et al. 2021). One potential explanation for the discrepancies in the efficacy of flavonoids against AgNP is that the different sizes of AgNP may elicit neutrophils oxidative burst through distinct mechanisms. Whilst the majority of flavonoids were observed to inhibit the oxidative burst, diosmetin did not exhibit any effect, potentially due to the presence of a methoxy group in its structure (Rufino et al. 2021). In one in vivo study, Ali et al. (2022) highlighted the effects of two flavonoids, hesperidin and naringin in their glycoside form (at a dose of 100 mg/kg for 3 weeks after the AgNP), administered in mice. These compounds were evaluated for their impact on the production of RS and lipid peroxidation, induced by AgNP, ranging from 20 to 100 nm (at a dose of 100 mg/kg for one month). Both flavonoids were found to decrease the production of ^•^NO and the lipid peroxidation.

Furthermore, several studies have demonstrated that flavonoids possess antioxidant properties through alternative mechanisms, including the upregulation of antioxidant defences and the modulation of their expression or activity (Ahmed and Hussein 2017; Ali et al. 2022; Arisha et al. 2019; Elblehi et al. 2022; Goodarzi et al. 2023; Veisi et al. 2021). In vivo studies were conducted by Arisha et al. (2019) and Ahmed and Hussein (2017), employing two distinct animal models, Sprague–Dawley and Wistar rats, respectively, which were simultaneously treated for 8 weeks with AgNP and the flavonoids morin and rutin. The results of both studies demonstrated that the flavonoids morin and rutin, respectively, were able to induce an increase in the activity of several antioxidant enzymes, namely glutathione peroxidase (GP_X_), catalase (CAT) and SOD and antioxidant molecules, GSH (Ahmed and Hussein 2017). Elblehi et al. (2022) investigated the protective effect of quercetin (50 mg/kg bw per day) against the adverse effects of AgNP (50 mg/kg bw) both administered simultaneously to Wistar Albino rats for 30 days. The study demonstrated that quercetin significantly increased the expression of paraoxonase 2 mRNA (Elblehi et al. 2022), an intracellular enzyme crucial for protection against OS and the inflammatory effects (Campagna et al. 2024). Goodarzi et al. (2023) performed an in vivo study with male Wistar rats, pre-treated with quercetin at a dose of 100 mg/kg, administered 2 days before and 1 h prior to exposure to 100 mg/kg of 30–50 nm-sized AgNP for 30 days. The AgNP induced a significant increase in the expression of the NADPH oxidase 3 (NOX3). However, in the presence of the quercetin, a slight tendency to decrease the expression of NOX3 genes was observed, which in turn led to a decrease in RS (Goodarzi et al. 2023).

Only two studies have investigated the potential anti-inflammatory effects of flavonoids in response to AgNP-induced pro-inflammatory effects. These in vivo studies have demonstrated that quercetin attenuates the production of inflammatory mediators, particularly inflammatory cytokines, induced by AgNP (Elblehi et al. 2022; Goodarzi et al. 2023). A study that exemplifies this effect was conducted by Elblehi et al. (2022), which found that the administration of quercetin (at a dose of 50 mg/kg bw per day) simultaneously with AgNP (at a dose of 50 mg/kg bw) in Wistar Albino rats for 30 days provided a significant reduction on serum levels of IL-6 and showed a tendency to decrease the TNF-α levels (Elblehi et al. 2022). Interestingly, Elblehi et al. (2022) and Gooddarzi et al. (2023) employed the same flavonoid in their studies and both investigated the impact of quercetin on cytokines, where their results were achieved by examining the expression of genes responsible for cytokine production. However, Gooddarzi et al. (2023) used a different dose of flavonoid (100 mg/kg) and AgNP (100 mg/kg) administering quercetin 2 days before and 1 h prior to exposure of 30–50 nm-sized AgNP for 30 days. Elblehi et al. (2022) observed a significant reduction in the expression of TNF-α and IL-1β following the administration of quercetin. Similarly, Gooddarzi et al. (2023) demonstrated a notable decline in the expression of IL-6. Furthermore, Elblehi et al. (2022) revealed that quercetin modulated Nrf2 gene expression, increasing its activity. This, in turn, promoted the expression of antioxidant enzymes, helping to maintain redox homeostasis and regulate the inflammatory response. In this manner, the flavonoids demonstrated the ability to diminish the inflammatory mediators and upregulated the antioxidant defences, thereby reducing the pro-inflammatory effects induced by the AgNP (Elblehi et al. 2022).

Another crucial mechanism by which flavonoids counteract AgNP-induced toxicity involves preserving cellular viability and preventing apoptosis. These aforementioned actions were observed through the evaluation of cytotoxicity, which allows for the assessment of viability loss, as well as the modulation of pro- and anti-apoptotic mediators. The majority of the in vitro studies have investigated the protective effects of flavonoids, by assessing their effects on the cytotoxicity induced by the AgNP (Essawy et al. 2021; Faedmaleki et al. 2016; Martirosyan et al. 2014, 2016; Rufino et al. 2021). Considering the evaluation of the cytotoxicity, Essawy et al. (2021) conducted an in vitro study in which a fish cell line, EPC cell, was exposed to 56.1 nm AgNP, at concentrations ranging from 0.5 to 4 μg/mL and, simultaneously, rutin at concentrations from 32 to 1024 μg/mL. It was observed that AgNP, per se, induced an increase in cytotoxicity, evidenced by the loss of cell viability (Essawy et al. 2021). However, in the simultaneous presence of the rutin, no cytotoxicity was observed. Similarly, Faedmaleki et al. (2016) exposed a primary culture of hepatocytes to 121.7 μg/mL of 20–40 nm-sized AgNP and simultaneously to silymarin at a concentration of 50–720 μg/mL, where the presence of the flavonoid promoted a decrease in cytotoxicity (Faedmaleki et al. 2016). Martirosyan et al. (2014) using Caco-2 cells simultaneously exposed, to 30–90 μg/mL of < 20 nm-sized AgNP, and quercetin or kaempferol, at concentrations of 10–50 µM, demonstrated the potential of this flavonoids in reducing cytotoxicity. In the in vitro study carried out by Rufino et al. (2021) using Caco-2 cells, simultaneously exposed to 4–19 nm PEI-AgNP (at a concentration of 5–50 µg/mL) and flavonoids (at a range of concentrations of 0–25 µM), quercetagetin and quercetin were the most effective compounds counteracting the cytotoxicity induced by PEI-AgNP. This research also investigated the ability of quercetin and quercetagetin (at a range of concentration of 3.13–50 µM) to modulate pro-apoptotic effects induced by PEI-AgNP. Both flavonoids demonstrated effects with regard to cell death; however, it is noteworthy that the flavonoids exhibited opposite effects. Whilst quercetin was able to decrease the apoptotic cells, quercetagetin had an opposite effect, appearing to increase the apoptotic cells, especially in the presence of 4 nm AgNP (Rufino et al. 2021), suggesting that this flavonoid exerts a synergistic effect with AgNP under study. With regard to in vivo studies, only two studies have demonstrated that different flavonoids (quercetin and morin) could attenuate AgNP-induced cell death through modulation of anti- and pro-apoptotic mediators. Arisha et al. (2019) reported that morin was able to downregulate mRNA expression of Bax, caspase-3, Fas, and FasL [mediators of programmed cell death (apoptosis)], whilst also increased Bcl-2 (anti-apoptotic mediator) and PCNA (proliferation marker) in Sprague–Dawley rats exposed for 8 weeks to AgNP (at a dose of 50 mg/kg bw per day) and morin (at a dose of 30 mg/kg bw per day) simultaneously (Arisha et al. 2019). Additionally, Elblehi et al. (2022) demonstrated that the concomitant administration of quercetin (at dose a of 50 mg/kg bw per day) was capable of counteracting the effects of AgNP (at dose a of 50 mg/kg bw), in Wistar Albino rats by modulating the expression of the same mediators as morin, specifically pro-apoptotic proteins (Bax mRNA) and anti-apoptotic mediators (Bcl-2) (Elblehi et al. 2022).

The flavonoids also exhibit the capacity to preserve cellular integrity and epithelial barriers. This is achieved by enhancing the regulation of tight junctions, particularly through the modulation of mRNA expression. In the in vitro study conducted by Martirosyan et al. (2014), an enhancement in the molecular organisation was observed, resulting in the restoration of the protein distribution, particularly of occludin and zonula occludens-1 (ZO-1) affected by the AgNP. This was achieved through the simultaneous exposure to < 20 nm-sized AgNP (at a concentration from 30 to 90 μg/mL) and the flavonoids, namely quercetin and kaempferol (at concentrations from 10 to 50 µM), with quercetin exhibiting the most pronounced effect. The structural difference between these flavonoids is attributed to the presence of a catechol group (3’ and 4’ positions) at the B ring in quercetin, and a hydroxyl group in 4’ position of the B ring in the kaempferol. This difference may be responsible for the more pronounced effects of the quercetin. In a subsequent study conducted by the same author (Martirosyan et al. 2016), Caco-2 cells simultaneously exposed to <20 nm-sized AgNP (at a concentration of 15-90 /mL) and quercetin (at a concentration of 50 µM), a recover of lysosomal integrity was observed. Sirotkin et al. (2021) investigated the effects of 150 nm AgNP exposure on porcine granulosa cells. The cells were exposed simultaneously to AgNP and quercetin at concentrations ranging from 0.1 to 100 µg/mL and 10 µg/mL, respectively. The results demonstrated that quercetin was unable to maintain the structural integrity of the monolayer (Sirotkin et al. 2021). This also indicates that the flavonoids protective effect may be contingent upon the specific experimental conditions under which they are tested. Arisha et al. (2019), using Sprague–Dawley rats exposed to 20 nm AgNP (at a dose of 50 mg/kg bw per day), and morin (at a dose of 30 mg/kg bw per day) simultaneously for 8 weeks, demonstrate that morin was able to improve the mRNA expression of claudin-11, occluding, and also the expression of transforming growth factor beta-1 (TGF-β1) (Arisha et al. 2019). In addition, in other in vivo study, Elblehi et al. (2022) using Wistar Albino rats exposed to AgNP (at a dose of 50 mg/kg bw) demonstrated that quercetin (at dose of 50 mg/kg bw per day) also improved the expression of tight junctions more specifically claudin-5. Goodarzi et al. (2023) studied the expression of proteins in Wistar rats pre-treated with quercetin (at a dose of 100 mg/kg) 2 days before and 1 hour prior exposure to 100 mg/kg of 30–50 nm-sized AgNP for 30 days. The observed decrease in protein expression in the cochlea was interpreted as a potential indicator of tissue damage. The authors reported that the quercetin also had the ability to increase the genes expression of two types of proteins that exist in ganglion cells: β-tubulin III (TUBB3) (Goodarzi et al. 2023), a component of microtubules that represents a significant component of the cell cytoskeleton (Duly et al. 2022), and myosin heavy chain VII (MYH7a) protein (Goodarzi et al. 2023), a mechanoenzymes that converts ATP to mechanical forces moving along actin filaments (Coffin et al. 2007; Marian and Braunwald 2017).

Additionally, only in in vivo studies, it was also demonstrated that even different flavonoids were able to reduce the damage in tissue whilst maintaining their architecture and functions. This protective effects were observed through the presence of biomarkers indicative of hepatic damage, namely, alanine aminotransferase (ALT), aspartate aminotransferase (AST), alkaline phosphatases (ALP), blood urea nitrogen (BUN), γ-glutamyltransferase (GGT), bilirubin, uric acid, and creatinine, which are present in the blood (Ali et al. 2022; Arisha et al. 2019; Veisi et al. 2021), as well as morphological alterations (Ali et al. 2022; Arisha et al. 2019; Goodarzi et al. 2023). Ali et al. (2022) demonstrated that the exposure for 3 weeks of hesperidin and naringin (at a dose of 100 mg/kg) after 1 month of exposure to the 20–100 nm AgNP (at a dose of 100 mg/kg), effectively counteracted hepatic tissue damages, in mice. This protective effect was evidenced by a reduction in biomarkers, such as AST, ALT, ALP, GGT, and bilirubin (Ali et al. 2022). Both flavonoids demonstrated capability to restore the hepatic architecture and decreased the pathological alterations, such as diffuse hydropic degeneration and vacuolation congested sinusoids (Ali et al. 2022). Arisha et al. (2019), using Sprague–Dawley rats, simultaneously exposed to morin (at a dose of 30 mg/kg bw per day) and 20 nm AgNP (50 mg/kg bw per day) for 8 weeks, demonstrated that morin had the capacity to reduce the damage caused by the AgNP in testicular tissue, restoring the sperm parameters and decreasing the serum levels of hepatic biomarkers (e.g., ALT, AST ALP, BUN, uric acid, and creatinine) (Arisha et al. 2019). Goodarzi et al. (2023) also found that the administration of quercetin (100 mg/kg), in Wistar rats before the exposure to 30–50 nm AgNP, mitigated histological changes. Quercetin showed capability to substantial improved the loss of hair cells (HC) and spiral ganglion cells (SGN); furthermore, the quercetin was able to regenerate the cells responsible for hearing (Goodarzi et al. 2023).

In addition to the aforementioned protective effects, flavonoids have also been observed to possess more specific actions, including the ability to restore the balance between hormone levels (Arisha et al. 2019; Sirotkin et al. 2021). Arisha et al. (2019), reported a modulation in the hormonal mRNA expression in Sprague–Dawley rats treated concomitantly, for 8 weeks, with 20 nm AgNP (50 mg/kg bw per day) and morin (30 mg/kg bw per day). The presence of morin alleviated this decrease by triggering the mRNA expression of hypothalamic gonadotropin-releasing hormone (GnRH1), testicular androgen (AR), luteinizing hormone (LH), and free testosterone. However, on follicle-stimulating hormone (FSH) and total testosterone, only a slight increase was observed (Arisha et al. 2019). Controversially, in an in vitro study, Sirotkin et al. (2021) using porcine granulosa cells exposed to < 150 nm AgNP (at a range of concentration of 0.1–100 µg/mL) and quercetin (at a concentration of 10 µg/mL), simultaneously, demonstrated that quercetin did not have ability to inhibit progesterone release on ovarian cell (Sirotkin et al. 2021).

Only in two in vivo studies, conducted by Ahmed and Hussein (2017) and Elblehi et al. (2022), focussed on the effects of rutin and quercetin, against AgNP-induced neurotoxicity, in Wistar and Wistar albino rats, respectively (Elblehi et al. 2022; Ahmed and Hussein 2017). Elblehi et al. (2022) using Wistar Albino rats exposed simultaneously to AgNP (at a dose of 50 mg/kg bw) and quercetin (at dose of 50 mg/kg bw per day) reported that the quercetin induces an increase on the levels of gamma-aminobutyric acid (GABA), an inhibitory neurotransmitter and also verified an increase in acetylcholinesterease (AchE), a naturally neurotransmitter that is a cholinergic enzyme that breaks down or hydrolyses acetylcholin and is considered a potential biomarker of xenobiotic toxicity. The authors also denoted that AgNP also induced histological abnormalities in the cerebrum and cerebellum. The concomitant administration of quercetin attenuated these damages (Elblehi et al. 2022). Furthermore, Ahmed and Hussein (2017) research simultaneously exposed Wistar rats, for 8 weeks, to < 100 nm PVP-coated AgNP (at a dose 30 mg/kg bw per day) and rutin (at a dose 50 mg/kg bw per day), was a more extensive investigation which assessed its impact on neurotransmitters, receptors, and neuronal enzymes, thereby demonstrating the capacity of rutin to regulate these to control levels (Ahmed and Hussein 2017). In this study, the authors demonstrated that rutin was able to decrease the levels of excitatory amino acids neurotransmitters (glutamate and aspartate), increase the levels of inhibitory amino acids neurotransmitters (GABA and glycine) and other neurotransmitters, the dopamine, serotonin, nor-epinephrine which are biogenic amines, decrease the expression levels of genes related to N-methyl-D-aspartate (NMDA) receptors, glutamate receptors which is a primary excitatory neurotransmitter, and transcriptional levels of mRNA of monoamine oxidases (MAO)-A, MAO-B, that catalyses the oxidative deamination of biogenic amines and metallothionein (MT)-III. Considering the morphological aspects, the authors reported histological changes in brain tissue, namely, severe congestion, oedema and haemorrhage of blood vessels with neural degeneration and vacuolation, and astrogliosis and demyelination of neurons caused by < 100 nm AgNP. However, the presence of rutin demonstrated an improvement in these histological alterations (Ahmed and Hussein 2017).

Although the studies were conducted under different experimental conditions—such as different concentrations/doses of flavonoids and AgNP, presence or absence of coatings, incubations times, and models—most reached a common conclusion: flavonoids can effectively mitigate AgNP adverse effects. Overall, the results summarised in Table 2 suggest that almost all flavonoids have protective properties, notably antioxidant, anti-inflammatory and cytoprotective activities, which helped counteract the adverse effects of AgNP exposure.

### Protective effect of flavonoids against AgNP-induced toxicity in modified-AgNP

Based on the findings that individually introduced flavonoids can attenuate the harmful effects of AgNP, there is growing interest in exploring the potential of flavonoids used in modified-AgNP using flavonoid as coatings, core–shells, or loaded in the nanoparticle to mitigate the adverse effects associated with AgNP exposure. The existing research in this area remains limited, with most studies focussing on the green synthesis of AgNP or on enhancing the biological effects of the AgNP (through the use of flavonoids) or flavonoids. It is, however, important to note that not all studies provide a comparison with AgNP alone, without flavonoids, which would allow a clearer assessment of the flavonoids potential to reduce adverse effects.

This section considers only three studies demonstrating that modified-AgNP using flavonoids can reduce induced toxicity. The flavonoids studied included quercetin (Kumawat et al. 2022), hesperidin (Ren et al. 2022), and isoorientin (Wang et al. 2021) each referenced in a single article. Interestingly, the modification of AgNP using flavonoids varied across studies: one study used quercetin as a coating (Kumawat et al. 2022), another loaded isoorientin into the AgNP (Wang et al. 2021), and a third employed hesperidin in a core–shell configuration (Ren et al. 2022). It is noteworthy, that the three studies were in vitro investigations using standard cell lines, including, human umbilical vein endothelial cells (HUVEC) (Ren et al. 2022), liver cells (HL-7702) (Wang et al. 2021), erythrocytes (Kumawat et al. 2022; Wang et al. 2021), and RAW 264.7 macrophages (Kumawat et al. 2022).

Two studies primarily examined the biological effects of modified-AgNP with flavonoids and also assessed the cytotoxic effects of the AgNP on different cell lines (Kumawat et al. 2022; Ren et al. 2022). Ren et al. (2022) evaluated the impact of modified-AgNP using hesperidin as a core–shell structure, to assess its effect on cytotoxicity. The author demonstrated that the 20 nm AgNP with hesperidin as core–shell, at a range of concentrations from 30 to 120 µM, exhibited reduced cytotoxicity when compared to the cytotoxicity induced by the AgNP, in HUVEC cells (Ren et al. 2022). Similarly, Kumawat et al. (2022) demonstrated that a different flavonoid in a different form in the AgNP yielded the same result. The authors used double-functionalized AgNP with curcumin and one of three compounds, namely, quercetin, tyrosine, or isoniazid. Their findings indicated that RAW 264.7 macrophages, exposed to AgNP coated with curcumin and quercetin, at concentrations from 0.00412 to 0.0824 mmol/L, exhibited reduced cytotoxicity when quercetin was used as the coating agent, compared to AgNP coated only with curcumin. This effect was concentration-dependent, with higher concentrations showing more significant cytotoxicity (Kumawat et al. 2022).

Additionally, in vitro studies evaluated the haemocompatibility of modified-AgNP with flavonoids*.* It is of great importance to ascertain the compatibility of AgNP with blood, given that blood represents a primary target for the toxic effects induced by AgNP when they are present in the body, assessing haemocompatibility is crucial (Kumawat et al. 2022; Wang et al. 2021). Wang et al. (2021) investigated the haemocompatibility of 117 nm AgNP loaded with isoorientin, at concentrations ranging from 60 to 240 μg/mL, demonstrating that isoorientin reduced haemolysis by lowering the percentage of haemolytic cells compared to AgNP without flavonoids. Interestingly, Kumawat et al. (2022) conducted a haemocompatibility assay on erythrocytes, using 32.71 nm AgNP double-functionalized with curcumin and quercetin. The authors observed that the presence of quercetin as coating in the AgNP resulted in a higher degree of haemolysis, in comparison to AgNP with a curcumin coating only. Notwithstanding the observed distinction between the AgNP with different coatings, a total haemolysis of less than 5% haemolysis was observed across all evaluated concentrations, thereby confirming the haemocompatibility of all AgNP employed, including those double coated with curcumin and quercetin (Kumawat et al. 2022).

Furthermore, in one study, the modified-AgNP using flavonoids demonstrated a capacity to protect against morphological cell changes. Wang et al. (2021) investigated the morphological effects in erythrocytes exposed to 117 nm isoorientin-loaded AgNP at concentrations from 60 to 240 μg/mL. AgNP alone induced irregular polygonal shapes and rupture in erythrocytes, whereas isoorientin appeared to mitigate these morphological alterations, as evidenced by a lower number of ruptured erythrocytes compared to AgNP alone.

Although research on the protective effects of flavonoids in modified AgNP remains limited, current evidence indicates that their use, regardless of form, whether as a coating, core–shell structure, or loaded component, may mitigate AgNP-induced cytotoxicity. Nevertheless, further research is required to reach more definitive conclusions on this topic.

### An overview of the synergistic interaction of AgNP and flavonoids in modulating biological effects

The effects of AgNP remain a subject of ongoing debate in the scientific community. Some authors claim that AgNP have the potential to elicit adverse consequences, such as the induction of OS or pro-inflammatory effects (Ferdous and Nemmar 2020; Kim and Ryu 2013; Sousa et al. 2022), whilst others suggest that AgNP may possess beneficial biological properties, such as antioxidant or anti-inflammatory effects (Bedlovičová et al. 2020; Carvalho-Silva and Reis 2024; Gherasim et al. 2020). Notably, as showed in Table 3, there has been a growing number of studies examining the biological potential of flavonoids in conjugation with AgNP, often focussing on the improvement of biological activities of AgNP and/or flavonoids when tested together, with a view to explore their potential for biomedical applications. These approaches have been shown to enhance the properties of AgNP and flavonoids, with particular focus on their antibacterial properties (Anwar et al. 2019; Badhwar et al. 2021; Hairil Anuar et al. 2024; Jackson and Dietrich 2024; Masri et al. 2021; Ren et al. 2022; Sun et al. 2023; Sun et al. 2017) as well as antiparasitic (Anwar et al. 2019; Siddiqui et al. 2020), antiviral (Krzyzowska et al. 2023), antifungal (Alqarni et al. 2022), anti-inflammatory (Elfaky et al. 2022; Kumawat et al. 2022; Rao et al. 2018), antioxidant (Elfaky et al. 2022; Kumawat et al. 2022), anticancer (Anwer et al. 2022; Bose et al. 2020), anticoagulant activities (Wu et al. 2020), and wound-healing properties (Badhwar et al. 2021; Ren et al. 2022).

**Table 3:**
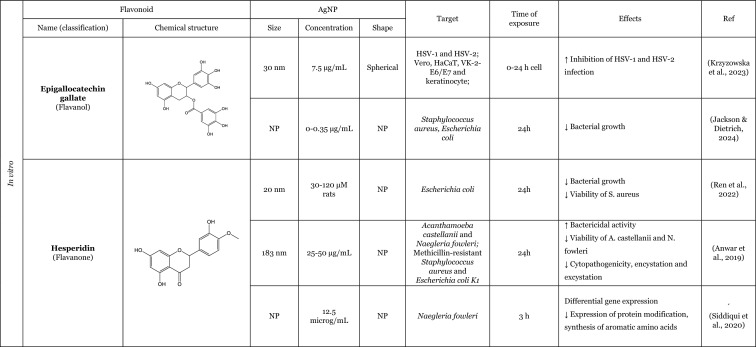

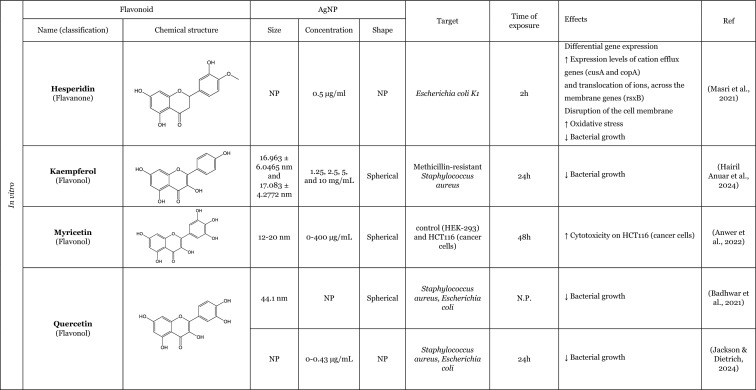

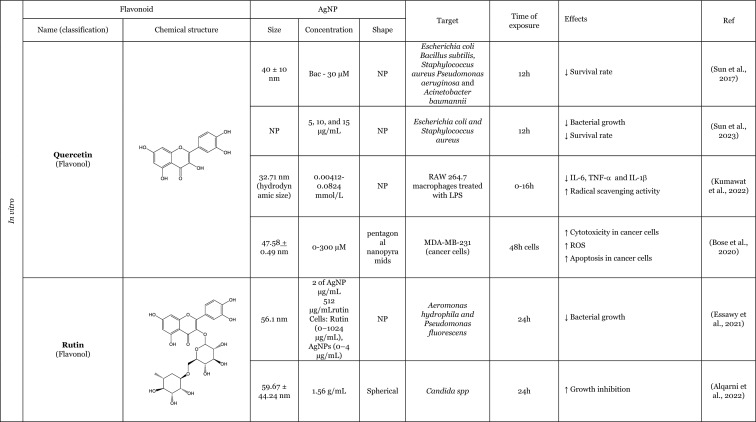

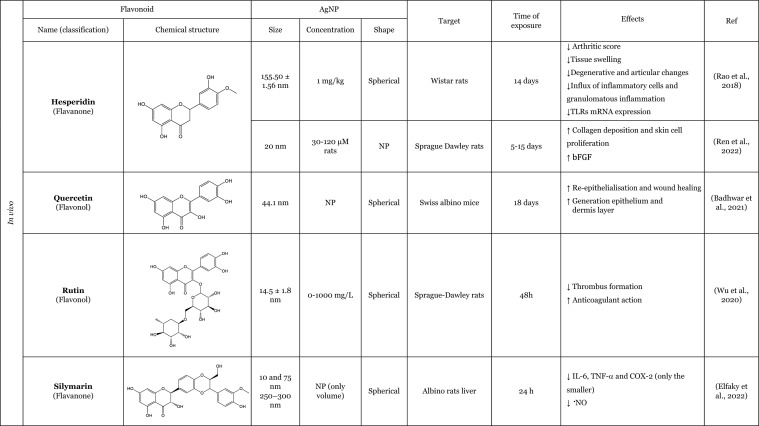
Overview of the in vitro and in vivo studies exploring the synergistic interaction of AgNP and flavonoids in improvement of biological effects

**↓** Decrease, **↑** Increase, *bFGF* Basic fibroblast growth factor, *COX*-*2* Cyclooxygenase-2, *IL*-*1β* Interleukin 1β, *IL*-*6* Interleukin 6, *NF*-*кB* Nuclear factor kappa B, *N*.*P*. Information not provided, ^•^*NO* Nitric oxide, *N*.*S*. Not significant, *PCNA* Proliferating cell nuclear antigen, *PVP* Polyvinylpyrrolidone, *ROS* Reactive oxygen species, *SOD* Superoxide dismutase, *TLRs* Toll-like receptors, *TNF*-*α* Tumour necrosis factor α

The majority of the studies have focussed on investigating the antibacterial activity. The AgNP possesses intrinsic antibacterial properties that are similar to those observed for flavonoids, which exhibit additional antimicrobial activity. Therefore, this combination represents an effective approach for the treatment of infectious diseases. The most commonly used bacterias for evaluating the antibacterial activity of the AgNP in combination with flavonoids were *Staphylococcus aureus* and *Escherichia coli*. However, the combination was also tested with *Aeromonas hydrophila, Pseudomonas fluorescens,* methicillin-resistant *Staphylococcus aureus, Escherichia coli K1, Bacillus subtilis,* and *Pseudomonas aeruginosa*. The combination of AgNP and flavonoids demonstrated an improvement in the inhibitory effect on bacterial growth (Badhwar et al. 2021; Essawy et al. 2021; Hairil Anuar et al. 2024; Jackson and Dietrich 2024; Ren et al. 2022; Sun et al. 2023), showed bacteriostatic/bactericidal activity (Anwar et al. 2019), decreased the survival rate (Sun et al. 2023, 2017), and also influenced gene expression, increasing the expression of ion translocation across the membrane genes and cation efflux genes (Masri et al. 2021). Interestingly, this antibacterial activity was not demonstrated against all bacteria, as proved by Sun et al. (2017). The author reported that AgNP combined with quercetin had no effect on some drug-resistant bacteria, including *Pseudomonas aeruginosa* and *Bacillus subtilis*. However, the combination was found to be effective against *Staphylococcus aureus* and *Escherichia coli,* demonstrating the ability to disrupt the cell wall and inhibit bacterial proliferation (Sun et al. 2017).

The antifungal effect has been also studied. The presence of flavonoids together with AgNP demonstrated the capacity to enhance another antimicrobial effect, namely the antifungal activity of AgNP coated with rutin against *Candida albicans,* one of the most causative pathogens responsible for fungal infections, resulting in the inhibition of fungal growth (Alqarni et al. 2022).

In addition to their antimicrobial properties, the antiviral potential of modified-AgNP with flavonoids has also been investigated. Krzyzowska et al. (2023) synthesised an AgNP functionalised with epigallocatechin gallate, to test its ability to treat HSV-1 and HSV-2. The antiviral potential was tested in three human cell lines: Vero and HaCat, as well as VK-2-E6/E7 cells, all of which had been infected with the virus. The epigallocatechin gallate-functionalised AgNP were more effective than the tannic acid-functionalised AgNP, which prevented infected cells from inhibiting virus penetration and viral attachment (Krzyzowska et al. 2023).

Concerning the antiparasitic activity, some studies revealed that the modified-AgNP with flavonoids enhance the antiparasitic action. The antiparasitic potential has been most extensively evaluated against *Naegleria fowleri*, the pathogen responsible for primary amoebic meningoencephalitis. Siddiqui et al. (2020) evaluated the effect of a modified-AgNP (loaded with hesperidin) against this parasite at the trophozoite stage. The authors reported that, *Naegleria fowleri* treated with AgNP conjugated with hesperidin showed a differential gene expression. Some of this genes were associated with OS response, DNA repair, cell division, cell signalling, and protein synthesis (Siddiqui et al. 2020). Anwar et al. (2019) investigated the protective effect of modified-AgNP with hesperidin (loaded) against not only this parasite but also against *Acanthamoeba castellanii*. These authors noted that AgNP loaded with hesperidin was more effective than AgNP per se, which exhibited a reduction in parasite viability and demonstrated the ability to inhibit encystation, which is responsible for drug resistance in *Acanthamoeba castellanii* (Anwar et al. 2019)*.*

Anticancer activity was another biological effect studied with modified-AgNP with flavonoids. In two studies, using cancer cell lines, namely HCT116 (Anwer et al. 2022) and MDA-MB-231 (Bose et al. 2020), the combination of flavonoids with AgNP, resulted in an enhancement of the anticancer activity, exhibiting an increase on the cytotoxicity against cancer cells (Anwer et al. 2022; Bose et al. 2020). In addition to increased cytotoxicity in these cells, Bose et al. (2020) also reported that the presence of quercetin in conjugation with the AgNP promoted an increase that the generation of ROS in cancer cells, which may be attributed to the anticancer activity of quercetin (Bose et al. 2020).

Furthermore, studies have showed that AgNP with flavonoids can also lead to an antioxidant effect. A study utilising AgNP that had been double functionalised with curcumin and quercetin demonstrated antioxidant properties, specifically through their capacity to scavenge radicals (Kumawat et al. 2022). In this study, the presence of the flavonoid in modified-AgNP promotes an improvement in the ability to scavenge 2,2-diphenyl-1-picrylhydrazyl (DPPH) radicals (Kumawat et al. 2022). Elfaky et al. (2022) using silymarin as a separately added flavonoid demonstrate the ability to reduce the ^•^NO production induced by lipopolysaccharides (LPS) (Elfaky et al. 2022).

Moreover, some studies have demonstrated that the presence of flavonoids with AgNP can enhance the anti-inflammatory effect, leading to a reduction in the expression of pro-inflammatory cytokines (IL-1β, TNF-α, and IL-6) (Elfaky et al. 2022; Kumawat et al. 2022) and the expression of cyclooxygenase-2 (COX-2), that promotes the conversion of arachidonic acid to prostaglandins (Elfaky et al. 2022) induced by LPS. To illustrate this action, Kumawat et al. (2022), co-treated RAW 264.7 macrophages with LPS and modified-AgNP (double functionalised with curcumin and quercetin). The presence of the quercetin promoted a better anti-inflammatory effect, reducing the transcription of pro-inflammatory cytokines genes, namely, IL-1β and TNF-α. In an in vivo study, the anti-inflammatory effect was also demonstrated on inflamed tissues in rheumatoid arthritis (Rao et al. 2018). Rao et al. (2018) showed an improvement of the anti-inflammatory effect of hesperidin loaded in AgNP, and this study was based on the premise that AgNP are preferred for use in the construction of drug nano-carrier systems due to their optical activity and surface-enhancing properties (Rao et al. 2018). The findings of this study indicate that the presence of flavonoids in conjunction with AgNP resulted in a reduction in tissue swelling, a decrease in degenerative changes, and a reduction in mild articular changes. Additionally, the study demonstrated a comparatively reduced influx of inflammatory cells and diminished granulomatous inflammation in ankle joint tissues, as well as a significantly decreased expression of Toll-like receptors (TLR) mRNA (Rao et al. 2018).

Furthermore, the impact of flavonoids together with AgNP on the healing of wounds was examined. The process of wound healing is complex and comprises four overlapping phases: homeostasis, inflammation, proliferation, and remodelling. The presence of the flavonoid together with AgNP was found to be an effective promoter of wound healing, promoting re-epithelialisation and the formation of a dermal layer (Badhwar et al. 2021), as well as increasing collagen deposition and skin cell proliferation (Ren et al. 2022). To illustrate this effect, Ren et al. (Ren et al. 2022) used hesperidin as the core–shell of the AgNP and reported that the presence of the flavonoid promoted a faster wound closure, better re-epithelialisation, better collagen deposition, and skin cell proliferation than AgNP. Subsequent tests revealed an elevated expression of basic fibroblast growth factor (bFGF), a protein involved in the acceleration of skin regeneration (Ren et al. 2022).

Overall, the combination of AgNP with flavonoids exhibits synergistic biological effects, particularly antimicrobial activity, highlighting its potential for biomedical applications.

## Conclusion

The use of AgNP has expanded exponentially in various sectors, making them an integral part of everyday life and leading to increased and constant human exposure. This widespread use has raised considerable concerns about their potential health impacts, as studies indicate that AgNP can induce adverse effects, including cytotoxicity, OS, pro-inflammatory effects, and cell death. Therefore, identifying compounds capable of countering these effects is crucial. Flavonoids, known for their wide range of biological activities—such as antioxidant and anti-inflammatory properties—show promise in mitigating the adverse effects induced by AgNP.

Current evidence suggests that flavonoids may offer protective effects against AgNP-related toxicity, whether used independently or in conjunction with AgNP. However, the extent of this protection is variable, probably due to differences in their structures, functional groups, and the way they are incorporated into AgNP (whether as a coating, core–shell, or loaded). Flavonoids exert this protection by providing antioxidant and anti-inflammatory effects, reducing cell death, and preserving cellular morphology. Amongst the flavonoids studied, quercetin stands out for its notable protective potential in both in vivo and in vitro studies. Nonetheless, further research is needed to clarify the mechanisms behind these protective effects to optimise the use of flavonoids in reducing AgNP toxicity.

## Data Availability

Not applicable.
